# Antifungal Activity of Novel Formulations Based on Terpenoid Prodrugs against *C. albicans* in a Mouse Model

**DOI:** 10.3390/pharmaceutics13050633

**Published:** 2021-04-29

**Authors:** Suvidha Menon, Xiuyi Liang, Richa Vartak, Ketankumar Patel, Antonio Di Stefano, Ivana Cacciatore, Lisa Marinelli, Blase Billack

**Affiliations:** 1 Department of Pharmaceutical Sciences, College of Pharmacy and Health Sciences, St. John’s University, 8000 Utopia Parkway, Jamaica, NY 11439, USA; menons@stjohns.edu (S.M.); liangx@stjohns.edu (X.L.); vartakr@stjohns.edu (R.V.); patelk@stjohns.edu (K.P.); 2 Department of Pharmacy, “G. D’Annunzio” University of Chieti-Pescara, 66100 Chieti Scalo, Italy; antonio.distefano@unich.it (A.D.S.); ivana.cacciatore@unich.it (I.C.)

**Keywords:** antifungal, *Candida albicans*, carvacrol, intravaginal formulations, minimum inhibitory concentration

## Abstract

Carvacrol (CAR), a phenolic monoterpenoid, has been extensively investigated for its antimicrobial and antifungal activity. As a result of its poor physicochemical properties, water soluble carvacrol prodrugs (WSCPs) with improved water solubility were previously synthesized and found to possess antimicrobial activity. Here, three novel CAR analogs, WSCP1, WSCP2, and WSCP3, were tested against fluconazole (FLU)-sensitive and -resistant strains where they showed greater antifungal activity than CAR against *C. albicans*. The probable mechanism by which the CAR prodrugs exert the antifungal activity was studied. Results from medium acidification assays demonstrated that the CAR and its synthetically designed prodrugs inhibit the yeast plasma membrane H+-ATPase (Pma1p), an essential target in fungi. In other words, in vitro data indicated that CAR analogs can prove to be a better alternative to CAR considering their improved water solubility. In addition, CAR and WSCP1 were developed into intravaginal formulations and administered at test doses of 50 mg/kg in a mouse model of vulvovaginal candidiasis (VVC). Whereas the CAR and WSCP1 formulations both exhibited antifungal efficacy in the mouse model of VVC, the WSCP1 formulation was superior to CAR, showing a remarkable decrease in infection by ~120-fold compared to the control (infected, untreated animals). Taken together, a synthetically designed prodrug of CAR, namely WSCP1, proved to be a possible solution for poorly water-soluble drugs, an inhibitor of an essential yeast pump in vitro and an effective and promising antifungal agent in vivo.

## 1. Introduction

Opportunistic fungal infections primarily caused by *Candida albicans* (*C. albicans*) are common. Despite being a commensal colonizer in various human niches (the oral mucosa, the gut, the vaginal tract, and the skin), *C. albicans* can successfully evolve as a pathogen because of its adaptability to the changing host microbiome (such as antibiotic treatment or when the host becomes debilitated or immunocompromised). The standard antifungal drug, fluconazole (FLU), is fungistatic in nature, and hence, is a concern to immunocompromised patients. The high mortality rates associated with systemic infection or blood-borne candidiasis is another growing concern. Altogether, the various evolved mechanisms to evade human immunity and the metabolic flexibility of *C. albicans*, the pharmacological limitations of antifungal drugs, and the growing emergence of FLU-resistant as well as multidrug-resistant strains in immunocompromised patients justifies the relevance of investigation of novel antifungal targets [1,2].

Essential oils are volatile, complex natural mixtures of either terpenes, terpenoids, aromatic, or aliphatic compounds formed by aromatic plants. Although the use of essential oils dates to ancient times, it is only recently that they have gained great attention because of their numerous effects in various fields like the pharmaceutical, food, sanitary, cosmetic, and perfume industries. Notably, these industries are adopting the use of green products to inhibit several pathogens as well as to render the product as fit to use. Extensive research has been conducted to understand the properties of essential oils, the structure–activity relationship (SAR) of constituents of essential oils, the mechanism of action, desirable and undesirable effects, etc. Various research studies have demonstrated that essential oils possess antibacterial, antifungal, and insecticidal properties [3,4,5,6]. Several in vitro studies have shown that phenolic compounds exhibit better antimicrobial activity compared to other components of essential oils [7,8]. 

**Figure 1:**
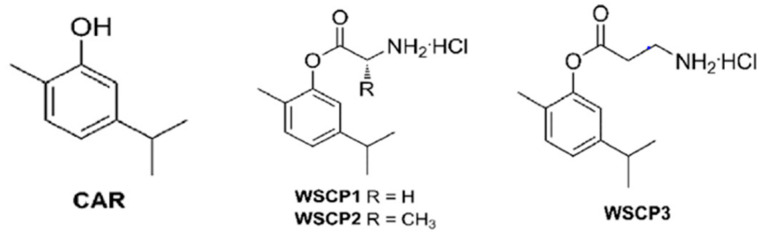
Chemical structures of CAR and its prodrugs.

Carvacrol (CAR, 2-methyl-5-(1-methylethyl)phenol), a phenolic monoterpenoid, is a major phytoconstituent of numerous aromatic herbs. Several insights into SAR studies have shown a link between physicochemical properties, such as hydrophobicity, the presence of a hydroxyl group, and a delocalized electron system, and carvacrol’s antimicrobial activity [7,9,10,11,12]. Research data has shown that the antifungal activity of CAR can be attributed to the inhibition of ergosterol biosynthesis and the disruption of membrane integrity, which is fungicidal. The commonly used drug FLU is fungistatic, although it acts by a similar mechanism to CAR [13]. Although CAR has been extensively studied, its poor physicochemical properties hinder its use therapeutically. Numerous water-soluble CAR prodrugs (WSCPs) and codrugs have been recently synthesized [9,14] to show better water solubility, thereby alleviating the bioavailability issues. In particular, in a previous study, following the prodrug approach, twenty-three CAR derivatives were prepared, and their physico-chemical and antimicrobial properties were evaluated. Among prodrugs, WSCP1–3 revealed the highest water solubility as well as antibacterial and antifungal properties against gram-negative bacteria and different species of *Candida*, respectively [14]. In the present study, both CAR and these three water soluble prodrugs (Figure 1) were investigated for activity against the yeast plasma membrane H+-ATPase (Pma1p), an essential pump in yeast that has been proposed to be a viable antifungal candidate drug target [15]. This was done using FLU-sensitive (S1) and FLU-resistant (S2) strains of *C. albicans*. 

Vulvovaginal candidiasis (VVC) is the most common infection occurring in women of childbearing age, which is caused by *C. albicans* [2]. A mouse model has been utilized to study vaginal yeast infections [16,17]. In the present study, our lab has repurposed CAR and its prodrug WSCP1 with an aim to maximize its intravaginal efficacy by developing locally delivered formulations of CAR and WSCP1 for a compatible in vivo delivery while retaining antifungal activity against the *Candida* strain used for VVC infection. Altogether, this is the first report that discusses novel formulations of CAR and WSCP1 as a possible intervention for VVC.

## 2. Materials and Methods

### 2.1. Chemicals, Cells, and Reagents

Dimethyl sulfoxide (DMSO) (cat# D1435) was obtained from the Sigma Aldrich Chemicals Company (St. Louis, MO, USA). The strain of *C. albicans* used in this study are sensitive to FLU (clinical isolate S1) and resistant to FLU (clinical isolate S2) and were kindly provided by Dr. J. Morschhäuser (University of Würzburg, Würzburg, Germany) [18]. CAR and its prodrugs WSCP1–WSCP3 were synthesized as previously described [9,14]. FLU (cat # B2094) was purchased from ApexBio (Boston, MA, USA). Captex^®^ 300 EP/NF (Glyceryl Tricaprylate/Tricaprate) was kindly gifted by Abitec Corp (Columbus, OH, USA). Kolliphor^®^ HS15 (PEG-15-Hydroxystrearate) was obtained as a gift sample from BASF (New York, NY, USA). A liquid yeast-extract, peptone and dextrose (YPD) medium (pH 6.5) was prepared by adding YPD media broth powder (50.0 g) from HiMedia Laboratories (cat # M1363; Mumbai, India) to distilled water (~1000.0 mL) along with adenine hemisulfate salt (0.4 g) from Sigma Aldrich Chemicals Company (cat # A9126; St. Louis, MO, USA) and then autoclaved at 121 °C for 15 min. YPD agar plates were prepared by the addition of 65 g of agar from Becton, Dickinson and Company (Franklin Lakes, NJ, USA; cat # DF0427-17-6) to 1 L of YPD liquid medium prior to sterilization. The molten mixture was then poured into petri dishes (20 mL/dish). Roswell Park Memorial Institute (RPMI) 1640 medium, buffered with 0.165 M MOPS (3-(N-morpholino) propanesulfonic acid) containing L-glutamine and lacking sodium bicarbonate, was purchased from Sigma-Aldrich Co. (cat # R6504; St. Louis, MO, USA). 96-well cell culture plates were purchased from Eppendorf (Hauppauge, NY, USA; Cat # 13-690-076).

### 2.2. Determination of Minimum Inhibitory Concentration (MIC) of Test Compounds (CAR and Its Prodrugs)

The two-fold broth microdilution method with small modifications following Clinical and Laboratory Standards Institute (CLSI) document M27-A guidelines was used to determine the MIC value [19,20]. The final inoculum suspension contained 1–5 × 10^5^ CFU/mL of yeast strains S1 or S2. The stock solutions of CAR, the prodrugs of CAR (WSCP1–3), and the FLU were dissolved in DMSO and diluted in RPMI 1640 medium to achieve the drug concentration (32 mM). A two-fold dilution of these test compounds was prepared in RPMI 1640 medium to obtain a concentration range of 16, 8, 4, 2, 1, and 0.5 mM. Triplicate samples were performed for each test concentration. Each test well was filled with 200 μL of the cell suspension followed by 200 μL of the appropriate 2×-concentration test compound. The concentration of DMSO in any well was ≤0.4% and did not affect yeast growth at this concentration. Two drug-free medium wells were employed to provide sterility and growth controls, whereas FLU served as a positive control. All plates were incubated at 30 °C for 48 ± 4 h, and MIC values were recorded following a visual observation of turbidity as compared to the drug-free growth medium. The MIC was defined as the lowest concentration showing complete inhibition of growth after 48 h in all three wells of a given treatment.

### 2.3. Determination of the Effect of Test Compounds (CAR and Its Prodrugs) upon Medium Acidification by S1 and S2 Strains of C. albicans

The medium acidification assay was performed as described previously [21,22]. Considering the MIC values, the test compounds (WSCP1, WSCP2, and WSCP3 along with CAR) were used in this assay to determine the inhibitory effect, if any, at an increasing concentration (0.1, 0.3, 1, and 3 mM) on the acidification of growth medium by S1 or S2 strains. The inhibitory activity of these compounds was compared to that of FLU, which was hypothesized to possess no activity against this plasma membrane pump. The concentration of test compounds required to inhibit medium acidification by 50% (IC_50MA_) was then determined from a plot of the change in pH at 30 min versus the concentration of the test compound and was compared with the results for untreated cells, which were assigned the value of 100%.

### 2.4. Preparation of CAR and WSCP1 Formulations

The CAR and WSCP1 formulations were prepared by using Captex^®^ 300 EP/NF and Kolliphor^®^ HS15 as solubilizing agents. For the CAR formulation, a mixture of 5% *v*/*v* carvacrol, 47.5% *v*/*v* Captex^®^ 300 EP/NF, and 47.5% *v*/*v* Kolliphor^®^ HS15 was made. For the WSCP1 formulation, 5% *w*/*v* of the WSCP1 powder was thoroughly mixed in a blend of 6.25% *v*/*v* Captex^®^ 300 EP/NF, 6.25% *v*/*v* Kolliphor^®^ HS15, and 87.5% *v*/*v* purified water.

### 2.5. In Vivo Antifungal Activity Assays Using CAR and WSCP1 Formulations

Animals: female BALB/c mice 18–22 g were purchased from Taconic Laboratories, Inc. (Albany, NY, USA). Mice were maintained in an Association for Assessment and Accreditation of Laboratory Animal Care (AAALAC)-approved Animal Care Centre (ACC) at St. John’s University (Jamaica, NY, USA). The study received the approval of the Institutional Animal Care and Use Committee (IACUC) of St. John’s University (Protocol # 1977). Animals were allowed to acclimatize for a week in the ACC. A previously reported mouse model of VVC was implemented here [23] with slight modifications; in particular, a clinical isolate, S1 yeast, was inoculated intravaginally.

(I) Growth conditions for *Candida albicans*: the S1 strain of *C. albicans* was grown in yeast-extract-peptone-dextrose (YPD) broth for ~11 h at 30 °C with shaking at 200 rpm to reach a stationary phase culture. Following incubation, the yeast culture was washed in sterile YPD and enumerated on a YPD agar plate to determine the colony forming units (CFUs/100 µL).
(II) Vaginal inoculation: The murine VVC study accounted for a total of 9 days (Supplementary Materials, Figure S1). Briefly, mice were administered with 0.2 mg of β-estradiol 17-valerate dissolved in 100 µL sesame oil by subcutaneous injection 72 h prior to inoculation (day-3) and on day 3. Estrogenized mice were intravaginally inoculated by introducing 20 µL of YPD-containing *C. albicans* S1 strain blastoconidia (5.5 × 10^5^ CFU/20 µL) into the vaginal lumen (day 0), and the infection was allowed to progress until day 6. After development of the VVC model, we stepped into the intervention study. The interventions were administered intravaginally on three consecutive days (days 3, 4, and 5). All the animals were infected and then treated with the respective treatments: blank, CAR (50 mg/kg), and WSCP1 (50 mg/kg). A 6th-day-infected group served as a control for the baseline value for infection. For further information about treatment groups, see Supplementary Figure S1 in the Supplementary Materials.
(III) Endpoints: Immediately after animals were euthanized (day 6), ~100 µL of vaginal lavage fluid was collected to determine the vaginal fungal burden, while the vaginal tissues were excised for histopathological analysis.
  (a) Determination of vaginal fungal burden
  The vaginal lavage fluid was serially diluted and plated on a YPD agar plate containing ampicillin (100 µg/mL). The YPD agar plates were then incubated for 48 h at 30 °C, and the colonies were counted manually. The data was plotted as log CFU/100 µL of vaginal fluid for each of the treatment groups.
  (b) Histological analysis of the vaginal tissue
  Following the lavage, the vaginal tract was excised longitudinally, washed twice with phosphate-buffered saline, and fixed in 10% neutral-buffered formalin. The tissues were dehydrated with a gradual increase in alcohol concentrations followed by xylene. The tissues were then embedded in paraffin in an orientation that allowed cross-sectional cutting into 5 μm thick sections. All the tissue sections were mounted on a poly-l-lysine-coated slide and stained with hematoxylin and eosin (H & E) as previously described [24]. The images were acquired using a Zeiss Axio Scope A1 microscope (Micro-Optics Precision Instruments, Fresh Meadows, NY, USA) with Zeiss Zen 2.3 software.

### 2.6. Statistical Analysis

The results reported here represent the geometric mean ± standard error of the mean (SEM) from at least three representative experiments. The data were analyzed by one-way ANOVA followed by Dunnett’s multiple comparison test using GraphPad Prism 8.0. Statistical significance was considered at *p* < 0.05.

## 3. Results and Discussion

Antimicrobial drug discovery has been significantly impacted because of the large number of diverse chemical scaffolds naturally occurring in plants. The antibacterial and antifungal activity of CAR, a phenolic monoterpenoid, has been documented in various literature [9,13,14], and CAR prodrugs are better candidates than CAR with respect to their physico-chemical properties (they are more water-soluble and less volatile) (Table 1). Additionally, the former possesses improved antimicrobial activity [14]. 

The prodrug approach of coupling with a suitable amino acid was used in the drug design of WSCP1–3 to increase the solubility. The WSCP1 was coupled with glycine, whereas the WSCP2 and the WSCP3 were coupled with L-alanine and β-alanine, respectively [14]. In the present study, CAR and the prodrugs were evaluated for the antimicrobial activity by the broth microdilution method. The MIC values of the WSCPs and the CAR against both S1 and S2 strains at 48 h exhibited the following trend in order of decreasing potency: WSCP1, WSCP2 > WSCP3 > CAR > FLU (Table 2).

In this study, WSCP1 was the most active prodrug against *C. albicans*, showing an MIC value equal to 2 mM (0.4 mg/mL) compared to that of FLU (>16 mM), a drug of choice for the prevention and treatment of candidiasis.

The antifungal activity of CAR has been attributed to its effect on ergosterol biosynthesis, thereby disrupting the membrane integrity in a manner similar to FLU [13]. However, the present study was an attempt to assess whether CAR and its derivatives inhibit medium acidification by yeasts by reducing the activity of the plasma membrane H+-ATPase pump (Pma1p). The essential proton pump Pma1p, which maintains the intracellular pH, is vital for yeast growth. In general, the intracellular pH in a yeast cell is maintained between 6.0 and 7.5 by Pma1p [25]. The fungal Pma1p extrudes protons from the inside of the yeast cell to the external environment by coupling ATP hydrolysis to ion transport, thereby making the extracellular medium acidic [15,26]. Mechanistic studies have shown the impact of other naturally occurring chemical compounds such as eugenol acting on the H+-ATPase pump [25,27]. Our study showed that CAR and WSCP1 inhibits proton pump extrusion by S1 and S2 strains with an IC_50_ concentration of approximately 2 mM (Table 3), which was similar to the MIC concentration. 

The effect of the test compounds on the acidification of the growth medium surrounding the S1 and S2 colonies was studied by incubating the cells with increasing concentrations (0.1, 0.3, 1, and 3 mM) of CAR and WSCP1 and assessing their inhibitory activity on acidification over a period of 30 min (Supplementary Figure S2). Further studies will help to shed more light on this proposed mechanism and other possible mechanisms of these compounds.

The data presented here with WSCP1–3, the hydrophilic derivatives of CAR with a slightly lower log P than that of CAR [14], have shown an improvement in the antifungal activity against both the S1 and the S2 strains of *C. albicans* in vitro. Furthermore, considering the hydrophilic and hydrophobic balance of these compounds via the prodrug approach could help them dissolve in the microbial membrane and impair ergosterol biosynthesis, which is significant for the integrity of the fungal membrane. 

Lastly, we have determined the potential efficacy and lower toxicity of CAR derivatives in lieu of frequently used antifungal drugs in a mouse model of VVC. VVC has always been a leading reason for women to visit a gynecologist at least once during their reproductive age. Owing to the rise in resistant strains, currently available antifungal treatments are unable to prevent recurrent VVC episodes, resulting in boundless discomfort which necessitates the investigation and identification of novel compounds with intriguing targets and antifungal efficacy.

For in vivo assessment, CAR and the most active prodrug, WSCP1, were formulated using various excipients such as Captex 300 and Kolliphor HS15 based on the previous preformulation studies with a view to achieve effective solubilization of hydrophobic drugs [28]. These excipients were previously used in a similar study by our lab as well as widely incorporated in a range of studies in the cosmetics and pharmaceutics domain. In vivo studies using these excipients did not exhibit reproductive health toxicity and skin irritation, thereby corroborating that these excipients are generally regarded as safe at the concentrations used here [29].

**Figure 2:**
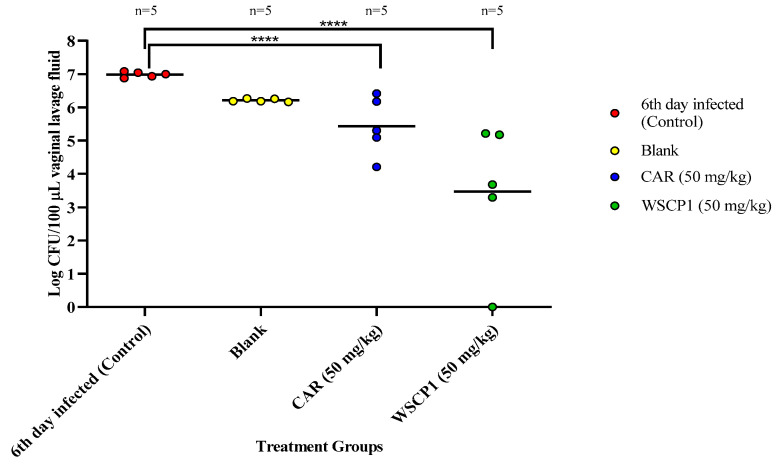
Effect of the CAR and the WSCP1 on VVC in a mouse model. **** *p* < 0.001 relative to control.

For implementing the mouse VVC model, estrogenizing the mouse is crucial since previous studies have shown a direct correlation of high estrogen levels with vaginal epithelium thickening and elevated glycogen content, resulting in increased susceptibility to VVC infection, which simulates the pregnancy or menstrual cycle conditions [2,23,30]. After the study period (day 6), mice were euthanized and vaginal lavage fluid was collected. The measurement of colony forming units (CFU/100 µL) exhibited the maintenance of a robust vaginal fungal burden by day 6 (Figure 2). 

The vaginal fungal burden indicated that the control group (infected, untreated) showed a high infection rate followed by blank, CAR (50 mg/kg), and WSCP1 (50 mg/kg). Treatment with the WSCP1 (50 mg/kg) formulation remarkably reduced the fungal burden by ~120-fold (Table 4). Mice treated with the CAR and the WSCP1 showed a high degree of variation in response to treatment, with one WSCP1-treated mouse showing a complete clearing of yeast. One could argue that this mouse represents an outlier point, but we do not believe it was an outlier. The mouse appeared healthy, and it was treated exactly like all the others in the cohort. There was no loss of vaginal lavage fluid or anything else that we observed that would lead to its exclusion from the study. While it remains unknown, the reason for the high variation in CAR- or WSCP1-treated mice is worthy of future study.

**Figure 3:**
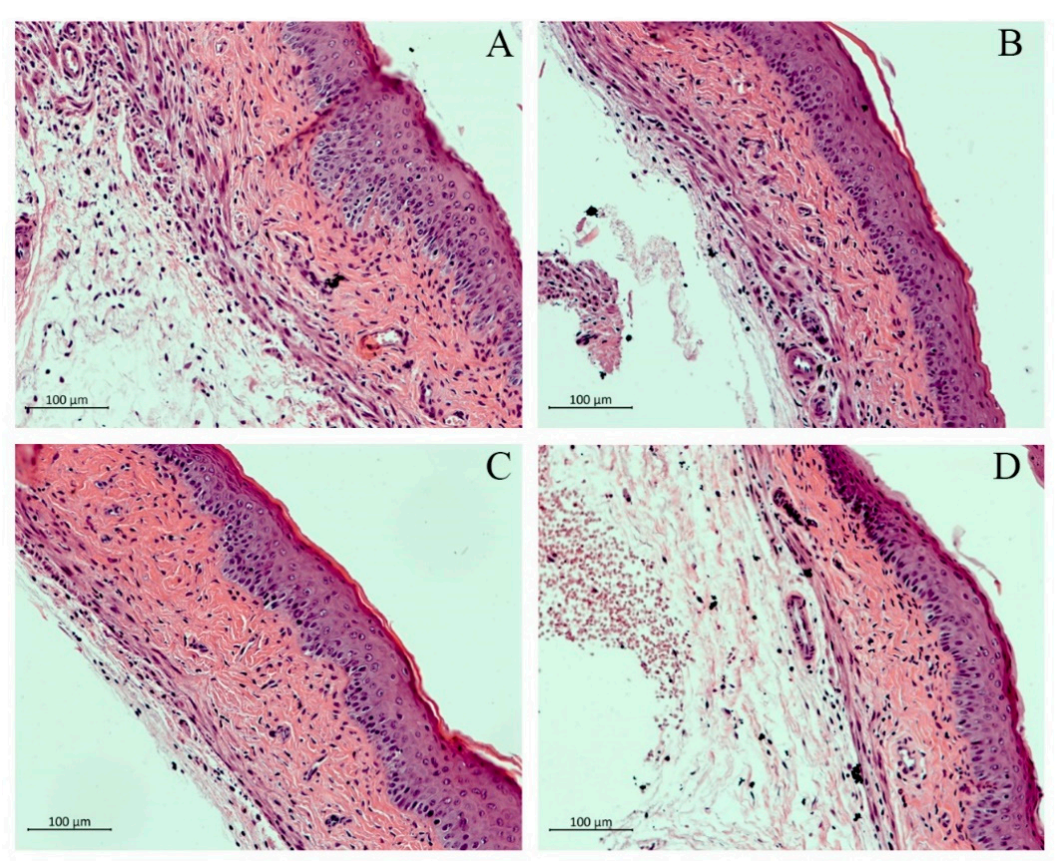
Histopathological analysis of vaginal tissues with H & E staining. Mouse vaginal tissue was excised longitudinally, fixed in 10% neutral-buffered formalin, and then embedded in paraffin. Each section of paraffin-embedded tissues was stained with H & E and then observed using alight microscope. (**A**) Naive group, (**B**) Blank, (**C**) CAR (50 mg/kg), and (**D**) WSCP1 (50 mg/kg). Magnification: 400×; scale bars, 100 μm.

This was further substantiated by the characteristic parameters of inflammation and damage to the vaginal mucosa such as vaginal epithelial cell (VEC) hyperplasia, edema, and damage to the keratin layer. H & E-stained vaginal tissues from the 6th-day group (infected, untreated) showed edema and hyperplasia of the VECs (Figure 3a), whereas the tissue sections from the blank-treated mice (Figure 3b) appeared healthy. Mice treated with CAR (50 mg/kg) (Figure 3c) and WSCP1 (50 mg/kg) (Figure 3d) showed slight epithelial distress (a loosely organized keratin layer) but otherwise appeared similar to blank-treated mice (Figure 3b). Further studies should be carried out to shed light on the formulation safety of CAR and WSCP1 to human vaginal epithelia.

Essential oils are aromatic in nature and derived from plants. They exhibit a broad spectrum of activities, including antifungal properties. At least one report has found that the susceptibilities of FLU-resistant strains of *C. albicans* to oregano, thyme, and ginger essential oils were higher than those of the FLU-susceptible yeasts [31]. More recently, transmission and scanning electron microscopy analyses of *C. albicans* treated with oregano oil inhibited both the growth and the activity of the FLU-resistant yeast strain *C. albicans* ATCC 14053 more efficiently than clotrimazole [32]. Nazzaro and colleagues (2017) have extensively surveyed the literature and have summarized the antifungal effects of essential oils such as membrane disruption, cell wall inhibition, toxicity to mitochondria, or inhibition to drug efflux pumps [33]. The present study focused on CAR, an important component of oregano oil, and its water-soluble prodrug WSCP1 and found that these compounds inhibit the yeast plasma membrane H+-ATPase. Whether this is due to a direct effect of these drugs on the ATPase itself or an indirect effect due to mitochondrial toxicity or membrane disruption is an area of interest for future study in our lab. It will be interesting to also determine the extent to which oregano oil itself inhibits the Pma1p of *C. albicans* and reduces the fungal burden in the mouse model of VVC.

The rationale for selecting the test dose of 50 mg/kg for the CAR and the WSCP1 in the mouse experiment was based on three previously published in vivo reports. First, an acute toxicity study in mice found that the LD10 and LD50 of carvacrol were 546.8 mg/kg and 919 mg/kg, respectively [34]. A second in vivo study in mice found that cognitive dysfunction associated with ethanol is alleviated by CAR at test doses of 50 mg/kg and 100 mg/kg [35]. Still yet another report found that CAR exhibited antinociceptive activity in mice at test doses of 50 mg/kg and 100 mg/kg [36]. Thus, we selected the 50 mg/kg test doses for the CAR and the WSCP1 used here based on these previous reports so that the test doses would fall well below the LD50 of CAR. Again, no such information is available for WSCP1 at the present time, but, since it is a CAR prodrug, we used the same test dose for both compounds.

The in vitro arm of the present manuscript also investigated the antifungal spectrum of CAR and its prodrugs by comparing the effects of these compounds in both an FLU-resistant clinical isolate (S2) and a matched FLU-susceptible isolate from the same patient (S1) [18]. Indeed, the mechanism of FLU resistance in the S2 clinical isolate has been described previously [37] and is derived from a gain-of-function mutation in the transcription factor Upc2p, over-expression of the transporter Mdr1p, elevated expression of nine genes involved in ergosterol biosynthesis, and an increased expression of a mutated ERG11 gene coding for a mutated endogenous Erg11p, wherein G307S and G448E substitutions decrease the affinity of the enzyme for FLU [37,38]. Moreover, overexpression of Erg11p in the S2 strain leads to the increased production of lanosterol demethylase, which contributes to azole resistance of this disease-associated strain [37]. In the present study, we have discovered that CAR and its prodrugs are effective as antifungal agents, at least in part, via a mechanism that involves Pma1p inhibition, even when the yeast strains exhibit elevated Mdr1p and Erg11p. Hosseini and colleagues (2016) found that CAR exhibited antifungal activity in clinical isolates of FLU-resistant *C. albicans* [39]; however, the extent to which Pma1p was inhibited by CAR in said isolates was not investigated. Thus, the present study adds another aspect to the antifungal mechanism of action of CAR, which is Pma1p inhibition by CAR and its prodrugs.

The IC_50MA_ values measured here serve as an index of the Pma1p inhibitory activity of CAR and WSCP1. Whereas the WSCP1 exhibited similar MIC (2 mM) and IC_50MA_ values (2.2 mM), indicating a concordance between Pma1p inhibition and antifungal growth inhibition, this was not the case for the CAR. In the case of the CAR, the MIC values were four-fold higher in the S1 (8 mM) and eight-fold higher in the S2 (16 mM) when compared to the IC_50MA_ values for the CAR in said strains (~2 mM). This indicates that while Pma1p inhibition by CAR is observed to occur and may contribute to the antifungal activity, additional mechanisms of fungal toxicity may be utilized for CAR, including disrupted ergosterol biosynthesis, increased membrane permeability and fluidity, and disrupted cationic gradients across the cell membrane [13,40,41]. CAR has also recently found to alter the cholesterol content of the HIV-1 viral membrane, blocking HIV-1 entry into target cells [42]. The extent to which WSCP1 can carry out these types of effects remains to be investigated but is anticipated, at least to some extent, based on its prodrug status and structural similarity to CAR.

## 4. Conclusions

Exploring novel targets in fungi may provide new pharmacological candidates that can combat the resistant strains. Our study was undertaken to understand if CAR and its prodrugs work by blocking the H+-ATPase pump (Pma1p), a viable target since it is critical to fungi survival. We extended our work from in vitro to in vivo by utilizing a novel intravaginal formulation of CAR and WSCP1, thereby alleviating the problem of drugs with poor water solubility. Furthermore, we identified the potential efficacy of CAR and WSCP1 as a therapeutic intervention in the mouse model of VVC.

## Figures and Tables

**Table 1:** Molecular properties of the CAR and the WSCPs.

Compounds	Molecular Weight (g/mol)	Water Solubility (mg/mL)	LogP *^a^*	Topological Polar Surface Area (TPSA) *^a^*
CAR	150.22	0.11	2.82	20.23
WSCP1	243.73	587	2.17	52.32
WSCP2	257.76	191	2.53	52.32
WSCP3	257.76	480	2.43	52.32

***^a^*** Prediction SwissADME platforms.

**Table 2:** MIC values for the CAR and its prodrugs and the FLU in the S1 and the S2 strains of *C. albicans* ^1^.

Test Compounds	Mean MIC_48h_	
	S1 Strain	S2 Strain
CAR	8 mM (1.2 mg/mL)	16 mM (2.4 mg/mL)
WSCP1	2 mM (0.4 mg/mL)	2 mM (0.4 mg/mL)
WSCP2	2 mM (0.5 mg/mL)	2 mM (0.5 mg/mL)
WSCP3	4 mM	4 mM
FLU	>16 mM	>16 mM

^1^ Unless otherwise indicated, all the MIC values represent the average of three experiments, each performed in triplicate.

**Table 3:** Pma1p inhibition by the CAR and the WSCP1 in the S1 and the S2 strain of *C. albicans.*

Test Compounds	Mean IC_50MA_ (mM)	
	S1 Strain	S2 Strain
CAR	2.0 ± 0.2	1.9 ± 0.6
WSCP1	1.6 ± 0.7	2.2 ± 0.4

**Table 4:** Treatment groups with fold reduction compared to the control group.

Treatment Groups	Fold Reduction Compared to the Control
6th-day-infected (control)	1.0
Blank	4.8
CAR (50 mg/kg)	8.8
WSCP1 (50 mg/kg)	122.2

## Data Availability

Data is contained within the article and supplementary material.

## Supplementary Materials

The following are available online at https://www.mdpi.com/article/10.3390/pharmaceutics13050633/s1. Figure S1: study design for intervention treatment of murine VVC; Figure S2: Medium acidification assay and inhibition of Pma1p by (**a**) CAR and (**b**) WSCP1.
