# Supplementary material for: Antifungal Activity of Novel Formulations Based on Terpenoid Prodrugs against C. albicans in a Mouse Model
Source: Pharmaceutics. 2021 Apr 29;13(5):633. doi: 10.3390/pharmaceutics13050633 (PMC8146751; doi:10.3390/pharmaceutics13050633)
Supplement: Supplementary file 1 [file pharmaceutics-13-00633-s001.zip › pharmaceutics-1160210-supplementary.pdf]

pharmaceutics

Supplementary Materials: Antifungal Activity of Novel Formulations Based on Terpenoid Prodrugs against *C. albicans* in a Mouse Model

Suvidha Menon, Xiuyi Liang, Richa Vartak, Ketankumar Patel, Antonio Di Stefano, Ivana Cacciatore, Lisa Marinelli and Blase Billack

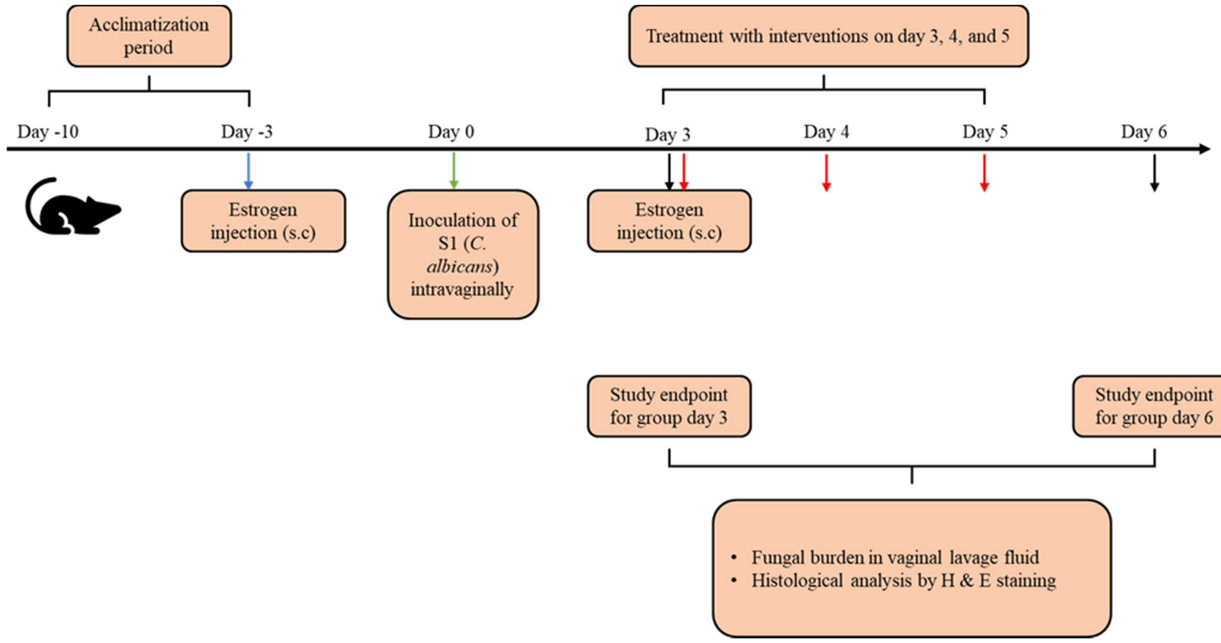

Figure S1. Study design for intervention treatment of murine VVC. Animals were acclimatized from Day -10 to Day -3 and were administered with s.c. injection of estradiol valerate except to naive group on day -3. On Day 0, animals were intravaginally inoculated with C. albicans S1 strain (5.5×10^5 CFU/20 µL); On Day 3, mice received a second subcutaneous injection of estradiol valerate except naive and mice from Day 3 group which were euthanized. Day 3 also marks the start of the intervention treatment to the respective treatment groups and were continued until Day 5. On Day 6, the remaining mice were euthanized, vaginal lavage fluid was collected, and the vaginal tissues were excised for further studies.

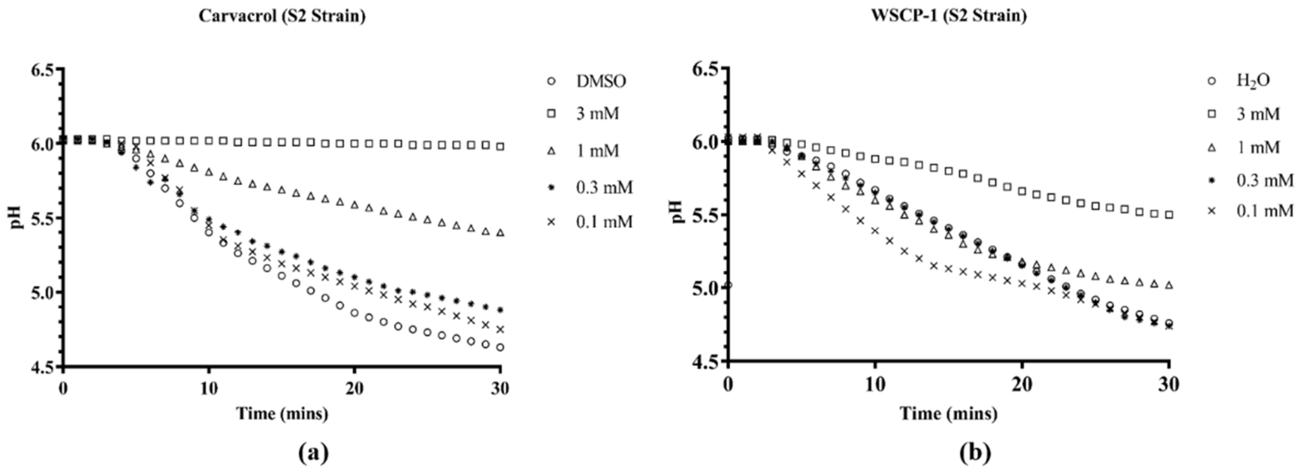

Figure S2. Medium acidification assay and inhibition of Pma1p by (**a**) CAR and (**b**) WSCP1.
